# Synovial Chondromatosis of the Ankle Joint: Clinical, Radiological, and Intraoperative Findings

**DOI:** 10.1155/2015/359024

**Published:** 2015-06-14

**Authors:** Sedeek Mohamed Sedeek, Q. Choudry, S. Garg

**Affiliations:** ^1^Department of Orthopaedics, East Lancashire Hospitals NHS Trust, Royal Blackburn Hospital, Blackburn BB2 3HH, UK; ^2^Department of Orthopaedics, Royal Lancaster Infirmary, Lancaster LA1 4RP, UK

## Abstract

Synovial chondromatosis, also termed synovial osteochondromatosis, is a rare benign disorder characterized by the presence of cartilaginous nodules in the synovium of the joints, tendon sheaths, and bursae. It most commonly involves large joints, such as the knee, hip, and shoulder, but its presence in smaller joints has also been reported. Nevertheless, ankle involvement is unusual. The diagnosis is commonly made following a thorough history, clinical, physical, and radiographic examination. We report a case of a young patient with primary synovial chondromatosis of the ankle joint and present the clinical, radiographic, and intraoperative findings.

## 1. Introduction

Synovial chondromatosis is a rare benign condition characterized by the formation of intra-articular cartilaginous nodules in the synovium of joints [1]. These nodules can detach and become loose bodies within the joint and may undergo secondary calcification and proliferation [2]. The etiology of synovial chondromatosis is not recognized. Histologically, synovial cells undergo metaplasia to chondrocytes, producing multiple cartilage nodules [3]. Synovial chondromatosis is most common in men in the third to fourth decade of life, often occurring in large joints, including the knee and hip, with smaller joints being less frequently involved [4, 5]. Patients commonly present with pain, swelling, and limited motion. Additionally, effusion, diffuse tenderness, and crepitus can be found on clinical examination [2].

Synovial chondromatosis originating from the ankle is an exceedingly rare condition [6, 7]. Herein, we present a case of synovial chondromatosis of the ankle joint treated by surgical excision and its outcome.

## 2. Case Report

A 21-year-old male patient presented to our clinic with 18-month history of pain in his left ankle which had deteriorated over the last year. He complained of stiffness, crepitation, and a catching sensation in the ankle; he felt as if he was “walking on pebbles.” Physical examination revealed a mildly tender swelling on the anterolateral aspect of the ankle joint. The patient was able to dorsiflex the ankle up to 10°, while plantar flexion was possible up to 20°. Anterior positive impingement was noted, while no instability was detected. There were no vascular or neurological abnormalities in the ankle and laboratory studies were within the normal range.

**Figure 1:**
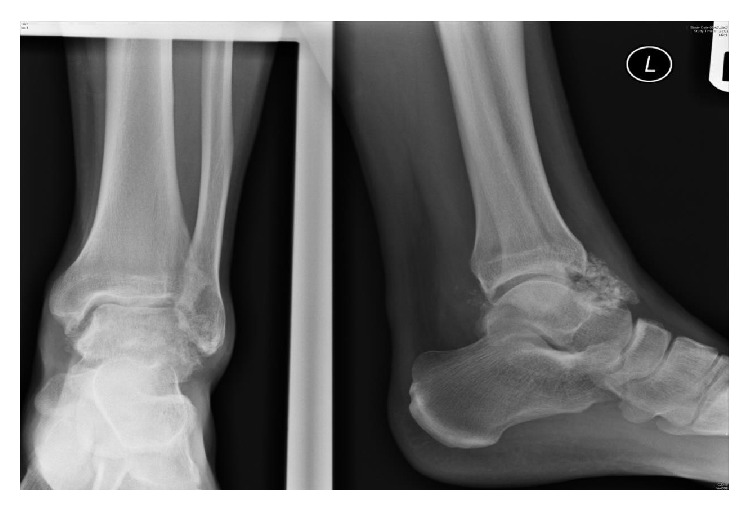
Preoperative radiographs showing a large speculated mass of calcified loose bodies.

**Figure 2:**
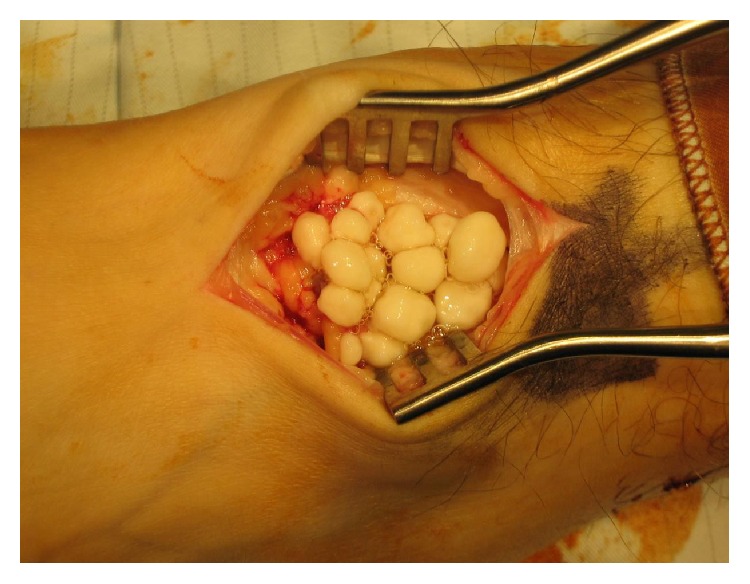
Arthrotomy of the ankle showing loose intra-articular bodies.

**Figure 3:**
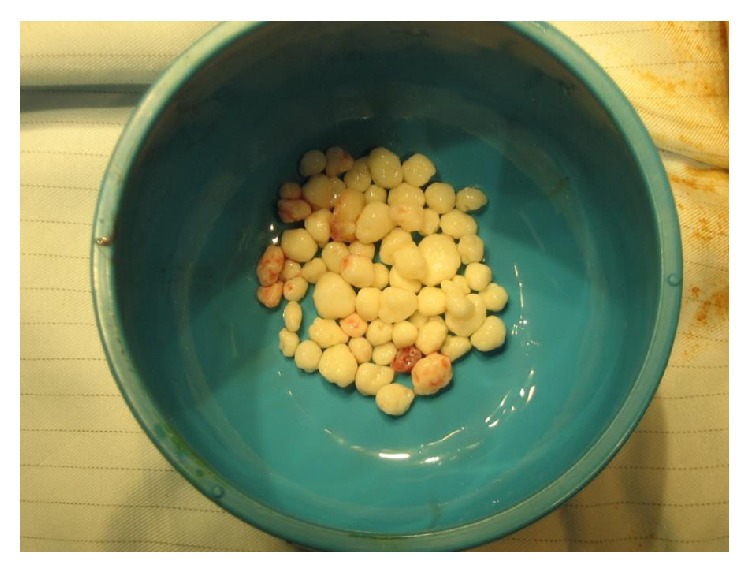
More than 40 retrieved loose bodies which were homogenous in appearance.

Plain radiographs of the left ankle revealed a large speculated calcified mass of loose bodies emerging from the anterior aspect of the ankle (Figure 1). As there was no history of direct ankle trauma or systemic inflammatory disease, primary synovial chondromatosis was strongly suspected. The patient underwent arthrotomy, surgical excision of the intra-articular bodies (Figure 2). Over 50 intra-articular bodies were retrieved, all of which were homogenous in appearance and ranged from 3 to 9 mm in size (Figure 3). The joint was irrigated by copious amounts of normal saline. There was no synovial proliferation or thickening; hence, a synovectomy was not performed. Postoperatively, the patient was allowed to perform partial weight bearing for 2 weeks. Histological examination confirmed the diagnosis of primary synovial chondromatosis. Postoperative radiographs showed no loose bodies in the ankle joint. At one-year follow-up, the patient was pain-free with a full range of ankle joint motion and had returned to his previous daily activity levels.

## 3. Discussion

Synovial chondromatosis is a rare benign synovial lesion characterized by multiple pearl-like intra- and extra-articular osteochondral loose bodies [1, 8]. Involvement is typically monoarticular, with large joints being most frequently affected. The knee joint is involved in 60 to 70% of cases and the shoulder, elbow, and hip are the next most frequently involved joints [9]. Synovial chondromatosis of the foot and ankle is a very rare condition. A review of the literature shows a limited number of reported cases involving the foot and ankle [10, 11].

It is broadly concurred that the exact etiology of synovial chondromatosis is unknown. Milgram et al. classified the disease process into three distinct stages [12, 13]. During the first stage, the synovial lining undergoes cartilaginous metaplasia. In the second stage, the nodules begin to detach from the synovium and appear as loose bodies; during this stage the patient becomes symptomatic. In the third stage, multiple loose bodies can be observed within the joint cavity with no visible intrasynovial bodies, indicating that activation of the synovium has subsided. The loose bodies also have a tendency to unite and calcify [12, 13].

Clinically, patients with synovial chondromatosis usually present with pain, swelling, stiffness, and/or a detectable mass, and most of them have a long clinical history before an accurate diagnosis is made [14]. There has been recent interest in the diagnosis of these cases due to a potential malignant degeneration. In 1998, Davis et al. reported a 5% relative risk for malignant degeneration in primary chondromatosis cases [15]. Imaging plays a crucial role in the diagnosis of synovial chondromatosis, with calcifications being present in standard radiographic examination in 70 to 95% of cases. Multiple calcified bodies, typically smooth, round, and of variable size, found within the joint capsule, are findings with diagnostic significance [16]. Nevertheless, radiographs may only reveal an increase in soft tissue density around the affected joint [7]. Thus, in cases where plain radiography cannot demonstrate calcification or ossification, magnetic resonance imaging is particularly useful [6].

The goal for the treatment of synovial chondromatosis is to remove the loose bodies, improve pain symptoms, regain movement in the joint, and limit the development of early osteoarthritis [7]. The treatment of choice is either open or arthroscopic surgical excision. Synovectomy is usually performed when active synovitis is present, usually stage 1 or stage 2. Most patients present in the late stage when active synovitis is no longer present; hence, a synovectomy is not required [17]. Recurrence occurs in 3% to 23% of cases, and it is thought to be after synovectomy with active synovium remaining or the presence of the stimulus which caused the metaplasia [5, 13, 18]. Our patient underwent open arthrotomy and loose body removal and was without recurrence at last follow-up. Open arthrotomy was the technique of choice to ensure complete removal of the heavy mass of calcified loose bodies observed in preoperative radiographic studies.

## 4. Conclusions

We report a case of primary diffuse synovial chondromatosis of the ankle. This unusual, distinctive benign synovial neoplasm presents readily recognizable clinical, radiographic, and pathological features. Primary synovial chondromatosis is adequately treated by loose body resection either arthroscopically or through open surgery. Prognosis is excellent.
